# First-in-human imaging and kinetic analysis of vesicular acetylcholine transporter density in the heart using [^18^F]FEOBV PET

**DOI:** 10.1007/s12350-020-02323-w

**Published:** 2020-09-09

**Authors:** Zacharie Saint-Georges, Vanessa K. Zayed, Katie Dinelle, Clifford Cassidy, Jean-Paul Soucy, Gassan Massarweh, Benjamin Rotstein, Pablo B. Nery, Synthia Guimond, Robert deKemp, Lauri Tuominen

**Affiliations:** 1 Department of Cellular and Molecular Medicine, University of Ottawa, Ottawa, ON Canada; 2 The Royal’s Institute of Mental Health Research, Ottawa, ON Canada; 3 Brain Imaging Centre, The Royal’s Institute of Mental Health Research, Ottawa, ON Canada; 4 Montreal Neurological Institute, McGill University, Montreal, QC Canada; 5 Department of Biochemistry, Microbiology, and Immunology, University of Ottawa, Ottawa, ON Canada; 6 University of Ottawa Heart Institute, Ottawa, ON Canada; 7 Division of Cardiology, University of Ottawa Heart Institute, Ottawa, ON Canada; 8 Department of Psychoeducation and Psychology, Université du Québec en Outaouais, Gatineau, QC Canada; 9 Department of Psychiatry, University of Ottawa, Ottawa, ON Canada

**Keywords:** Tracer development, molecular imaging agents, PET, molecular imaging, innervation tracers

## Abstract

In contrast to cardiac sympathetic activity which can be assessed with established PET tracers, there are currently no suitable radioligands to measure cardiac parasympathetic (cholinergic) activity. A radioligand able to measure cardiac cholinergic activity would be an invaluable clinical and research tool since cholinergic dysfunction has been associated with a wide array of pathologies (e.g., chronic heart failure, myocardial infarction, arrythmias). [^18^F]Fluoroethoxybenzovesamicol (FEOBV) is a cholinergic radiotracer that has been extensively validated in the brain. Whether FEOBV PET can be used to assess cholinergic activity in the heart is not known. Hence, this study aimed to evaluate the properties of FEOBV for cardiac PET imaging and cholinergic activity mapping. PET data were collected for 40 minutes after injection of 230 ± 50 MBq of FEOBV in four healthy participants (1 female; Age: 37 ± 10; BMI: 25 ± 2). Dynamic LV time activity curves were fitted with Logan graphical, 1-tissue compartment, and 2-tissue compartment models, yielding similar distribution volume estimates for each participant. Our initial data show that FEOBV PET has favorable tracer kinetics for quantification of cholinergic activity and is a promising new method for assessing parasympathetic function in the heart.

Sympathetic and parasympathetic systems in the heart are critical for adequate cardiac function. While sympathetic innervation of the heart is assessed routinely with positron emission tomography (PET) radiopharmaceuticals mimicking norepinephrine,^1^ there are currently no suitable radioligands to measure cardiac cholinergic (parasympathetic) activity. The human heart has both neuronal and non-neuronal cholinergic systems that account for cardiac parasympathetic activity.^2^,^3^ Failure of these cholinergic systems underlies a wide array of pathologies (e.g., chronic heart failure, myocardial infarction, and sympathetic hyperactivity-induced cardiac remodeling).^3^ For example, reduced parasympathetic activity has been identified as inherent to the autonomic imbalance displayed in chronic heart failure patients while adequate parasympathetic response is thought to offset the deleterious effects of hypertrophic sympathetic signalling.^2^,^3^ Activation of the parasympathetic nervous system has been linked to the pathogenesis of atrial fibrillation.^4^ Also, abnormal parasympathetic activity has been associated with ventricular arrhythmias in patients with prior sudden unexpected cardiac arrest.^5^ Having a PET radioligand that would be able to measure cholinergic activity could help to better predict prognosis in those medical conditions and would be an indispensable research tool to further our understanding of the role of cholinergic activity in a myriad of ailments since acetylcholine is ubiquitous and indispensable in many biological systems.

Cholinergic neurotransmission is dependent upon vesicular storage of acetylcholine by the vesicular acetylcholine transporter (VAT), thus making an ideal target for radioligand aimed at assessing cholinergic activity. In the brain, the quantity of VAT determines the capacity to release acetylcholine. Vesamicol and several of its derivatives bind preferentially to the VAT and act as a non-competitive inhibitor, resulting in the decrease of stimulated acetylcholine release.^6^ Radiolabelled vesamicols have been used as PET ligands to study cholinergic activity,^7^ however none has been validated to study human cardiac cholinergic function yet. Among vesamicol-derived tracers, research has shown that [^18^F]fluoroethoxybenzovesamicol ([^18^F]FEOBV) is the most promising radioligand.^8^ [^18^F]FEOBV has also been extensively validated in the central nervous systems of animals and humans as a marker of cholinergic innervation density.^7^ Compared to other vesamicols, [^18^F]FEOBV possesses desirable kinetics, high affinity for VAT (19.6 nM), low affinity for sigma-1 receptors and low sigma-1/VAT affinity ratio; is sensitive to physiological and pathological VAT density fluctuations; and releases relatively low quantities of free [^18^F] fluoride.^9^–^13^ These properties also make of [^18^F]FEOBV a potentially better cholinergic tracer than non-vesamicol alternatives. The most prominent of which is [^11^C]Donepezil,^14^ a tracer that inconveniently also has a potent affinity (14.6 nM)^15^ for sigma-1 receptors, that are also found in the heart.^16^,^17^ In contrast, [^18^F]FEOBV has negligible affinity for these receptors.^9^ Whether [^18^F]FEOBV PET could be used to assess cardiac cholinergic activity is not known. Thus, the objective of this study was to test if [^18^F]FEOBV could be a viable tracer for cardiac PET imaging and cholinergic activity mapping.

This investigation was approved by Health Canada and the The Royal Ottawa Health Care Group Research Ethics Board. Data are available from the authors upon request. Four healthy participants (1 female; Age: 37 ± 10.2; BMI: 25.1 ± 2.1) were recruited. Before entering the study, each participant signed a written informed consent form. All participants were non-smokers, did not have any known past or present heart disorders and gave a negative urine drug screen before the PET scan. An average dose of 230 ± 50 MBq of [^18^F]FEOBV was injected into a left antecubital vein as a bolus injection over 1 minute. PET data were collected for 40 minutes (first participant scanned for 30 minutes only) starting at injection using a Siemens Biograph mMR.

Dynamic datasets (framing 9 × 10 s, 3 × 30 s, 2 × 60 s, 7 × 300 s) were reconstructed using the vendor supplied 3D OP-OSEM algorithm (3 iterations, 21 subsets) with corrections applied for normalization, dead-time, decay, scatter and random events. A 4-mm Hann smoothing was applied post-reconstruction. Images were corrected for attenuation using a mu-map derived from a breath-held Dixon MR image acquired pre-injection. The dynamic PET images were analyzed using FlowQuant version 2.4, which allows for both automatic and manual sampling along the vertical long, horizontal long, and short axes of the left ventricle (LV) myocardium (see Figure 1). The regional time activity curves from the left ventricle were fitted with three different models: Logan graphical, 1-tissue compartment (1-TCM), and 2-tissue compartment (2-TCM).

**Figure 1:**
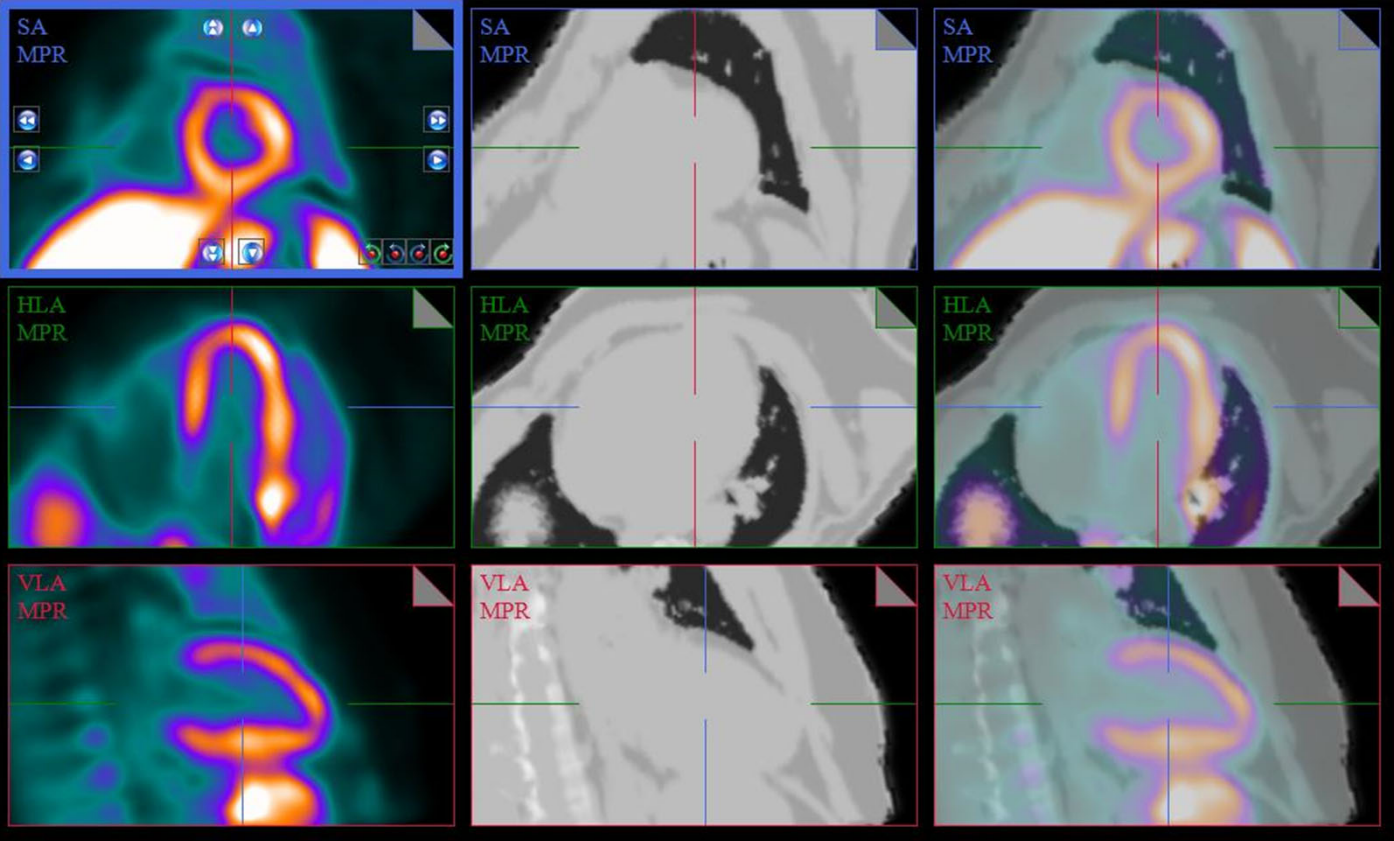
Fusion of cardiac PET and MR-derived attenuation images of subject B shown in short axis (SA), horizontal long axis (HLA), and vertical long axis (VLA) views

Consistently high [^18^F]FEOBV tracer uptake was observed in the left ventricle and time-activity curves quickly reached a plateau within the first 4 to 5 minutes, followed by a slow wash-out (Figure 2). 1-TCM, 2-TCM and Logan graphical analysis gave similar distribution volume (DV) estimates for each participant (Figure 3). Distribution volume estimated using the Logan graphical analysis model was 3.2 ± 0.6; using 1-tissue compartment model, 3.0 ± 0.6; and using 2-tissue compartment model, 2.9 ± 0.6. Furthermore, the DV estimates showed stability even when only the first 25 minutes post-injection were used in the analysis (Figure 3).

**Figure 2:**
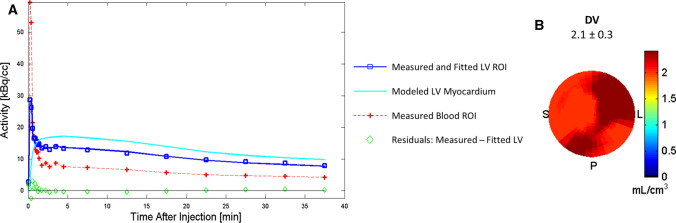
(**A**) Time-activity curves and fitted 1-TCM for subject B. (**B**) Polar Map (2D) of the [^18^F]FEOBV tracer distribution volume (DV) in the left ventricle (LV) myocardium of subject B

**Figure 3:**
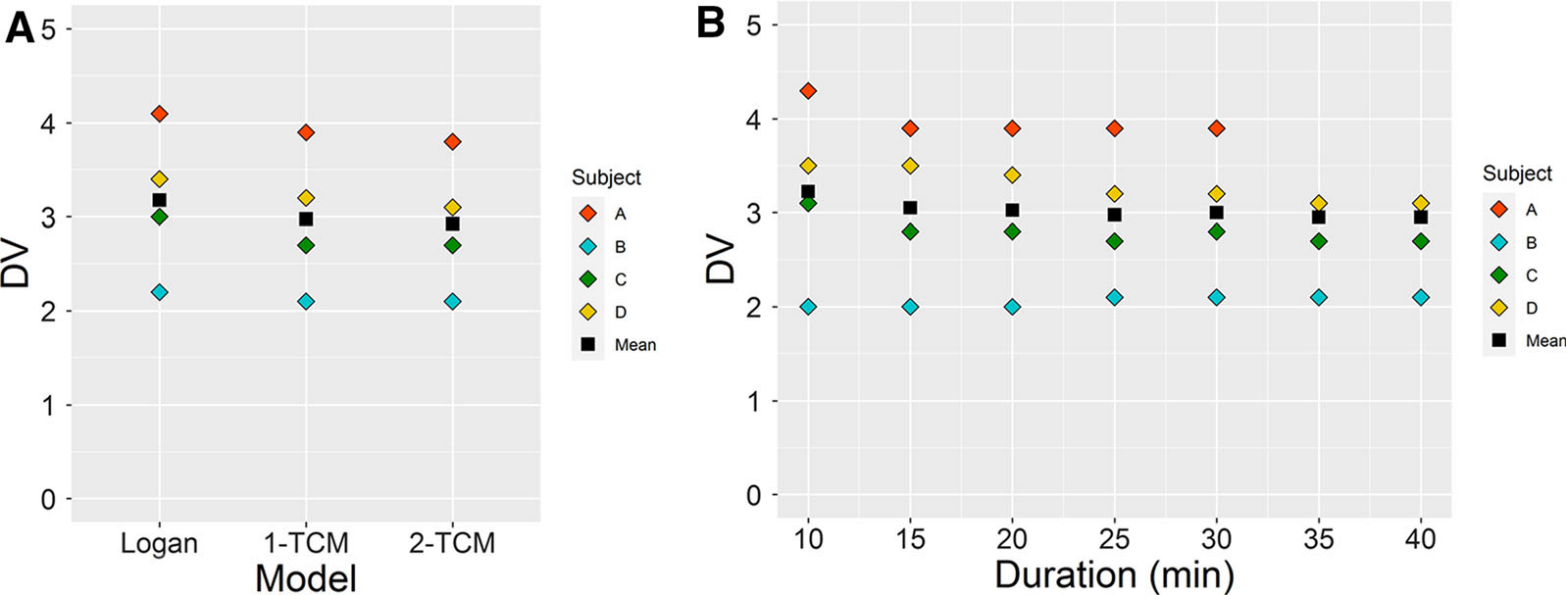
(**A**) Distribution Volume values per subject per model (Logan, 1-TCM, and 2-TCM) at 25 minutes of scan time. (**B**) Distribution volume values per subject for 1-TCM with different scan times

The [^18^F]FEOBV uptake and washout in the human heart were favourable for kinetic modelling, suggesting that [^18^F]FEOBV is a promising radiotracer to study the cholinergic systems in the heart. Since parasympathetic innervation is very limited in the ventricles,^18^ the fairly uniform distribution of this tracer throughout the left ventricle should mainly account for non-neuronal cholinergic handling in the myocardium.

The extent to which the radioactive metabolites may contaminate the signal in the heart remains unknown and is the main limitation of this study. As we did not collect blood samples during acquisition of PET data, we could not perform metabolite analyses or fit the PET data with metabolite corrected plasma activity. Because a total blockade of VAT would be toxic, [^18^F]FEOBV occupancy studies cannot be performed in humans to determine the specific binding fraction. Nonetheless, a prior report suggests that the fraction of [^18^F]FEOBV of the total radioactivity in plasma at 15 minutes post injection is above 70% but declines rapidly thereafter.^7^ Short scan durations should, to some extent, protect against the effect of blood metabolites. Our results suggest that a 25-minute scan time may be sufficient to estimate DV in the heart. Therefore, it would be feasible to image both the heart and brain from a single injection, since brain VAT DV can be estimated from a late static scan.^7^ Next step in the validation of this tracer will be to study patients known to have reduced cardiac cholinergic activity such as chronic heart failure patients.

The parasympathetic nervous system is involved in the pathogenesis of heart failure, myocardial infarction, atrial fibrillation and ventricular arrhythmias.^3^–^5^,^19^ Although imbalances within the autonomic nervous system have been demonstrated in patients with cardiac disease, non-invasive evaluation of parasympathetic activity has been limited. This is particularly important in patients with atrial fibrillation^19^ or those at risk for ventricular arrhythmias. While parasympathetic activation may trigger atrial fibrillation, current methods to evaluate the parasympathetic activity in humans are scarce. The main limitation of current studies evaluating parasympathetic activity has been the lack of accurate, reproducible diagnostic tools. Indirect measures of parasympathetic activity (e.g., heart rate variability^20^) are available, however these have limitations and need to be interpreted with caution due to a high susceptibility to confounding effects of age, sex, and comorbid conditions. Better understanding the effects of impaired cardiac cholinergic activity and reliable measurements of this activity could improve risk assessment and prognosis in cardiac patients. Furthermore, the availability of novel diagnostic tools may assist in the evaluation of therapeutic modalities targeting the parasympathetic nervous system to treat heart disease.^21^ Our initial data show that [^18^F]FEOBV PET is a promising new method for assessing the cholinergic systems in the heart.

## New Knowledge Gained

[^18^F]FEOBV appears to be a viable tracer for the assessment of cholinergic (parasympathetic) activity in the heart using non-invasive PET imaging.

## Conclusion

[^18^F]FEOBV PET is a promising radioligand to assess cholinergic activity in the heart and may become a useful non-invasive method to study cardiac parasympathetic function and improve risk assessment and prognosis in cardiac patients with conditions associated with autonomic imbalance or impaired parasympathetic activity.
